# SNHG17 Serves as an Oncogenic lncRNA by Regulating the miR-361-3p/STC2 Axis in Rectal Cancer

**DOI:** 10.3389/fgene.2021.654686

**Published:** 2021-06-23

**Authors:** Fuda Huang, Hua Li, Zebang Qin, Anmin Wang, Ya Zhang, Junyu Guo, Mingwei Wei, Houji Guo, Jian Pu

**Affiliations:** ^1^Proctology Department, Affiliated Hospital of Youjiang Medical University for Nationalities, Baise, China; ^2^Graduate College, Youjiang Medical University for Nationalities, Baise, China; ^3^Department of Hepatobiliary Surgery, Affiliated Hospital of Youjiang Medical University for Nationalities, Baise, China

**Keywords:** long non-coding RNA, SNHG17, miR-361-3p, STC2, rectal cancer

## Abstract

Long noncoding RNA (lncRNA) have been reported to be crucial regulators for carcinogenesis, including rectal cancer. This work aimed to explore the roles and associated mechanisms of small nucleolar RNA host gene 17 (SNHG17) in rectal cancer. A quantitative real-time polymerase chain reaction was performed to measure the expression level of SNHG17 in rectal cancer tissues and cells. Cell counting kit-8 (CCK-8) assay and flow cytometry assay were conducted to measure the biological roles of SNHG17 in rectal cancer. In addition, luciferase activity reporter assay, RNA immunoprecipitation (RIP) assay, and rescue experiments were conducted to explore the mechanisms of SNHG17 in rectal cancer. The upregulation status of SNHG17 was identified in rectal cancer tissues and cells. Functionally, knockdown the expression of SNHG17 inhibits rectal cancer cell proliferation *via* stimulating cell apoptosis. *In vivo* assay showed that the knockdown of SNHG17 inhibits tumor growth. Furthermore, we showed that microRNA-361-3p (miR-361-3p) has decreased expression in tumor tissues and cells, and SNHG17 functions as a sponge for miR-361-3p. The upregulation status of stanniocalcin 2 (STC2) was also found in rectal cancer, and the knockdown of STC2 hinders cancer progression. In conclusion, lncRNA SNHG17 functions as an oncogenic lncRNA in rectal cancer by regulating the miR-361-3p/STC2 axis.

## Introduction

Colorectal cancer (CRC) accounts for a large number of cancer-related deaths worldwide, each year (Siegel et al., 2016). Rectal cancer is a major subtype of CRC and can account for as much as 30% of CRC cases (Torre et al., 2012). The treatment methods for rectal cancer have been greatly advanced, but the survival rate remains at an unacceptable rate (Conde-Muíño et al., 2015). Hence, the exploration of molecular markers related to rectal cancer progression is important.

Non-coding RNAs (ncRNAs) are crucial regulators for cancer progression instead of “noise” in the genome (Anastasiadou et al., 2018). ncRNAs can influence various cellular biological processes through multiple mechanisms (Anastasiadou et al., 2018). Long non-coding RNA (lncRNA) is a type of ncRNA with limited or no capability to code proteins (Kung et al., 2013). Previous studies have indicated that lncRNAs may serve as important regulators for rectal cancer progression and development (Tao et al., 2018; Shao et al., 2019; Qu et al., 2020). For example, LINC00461 increased expression in rectal cancer cells and improved sensitivity to cisplatin through interacting with the microRNA-593-5p (miR-593-5p)/cyclin D1 axis (Qu et al., 2020). Another work showed that lncRNA small nucleolar RNA host gene 6 (SNHG6) expressed a high expression level in rectal cancer and correlated with shorter overall survival time (Shao et al., 2019). In addition, lncRNA NF-κB interacting lncRNA was downregulated in rectal cancer and correlated with advanced stage tumor (Tao et al., 2018).

Small nucleolar RNA host gene 17 (SNHG17) is a conserved lncRNA that exerts crucial roles in regulating cancer progression (Du et al., 2020; Liu et al., 2020). Recent studies have shown that SNHG17 could function as a competing endogenous RNA (ceRNA) in tongue squamous cell carcinoma and breast cancer (Du et al., 2020; Liu et al., 2020). However, the roles and regulatory mechanisms of SNHG17 in rectal cancer remain unclear.

In this work, we attempt to probe the roles and mechanisms of SNHG17 in rectal cancer, with the hope of providing a basis for the validation of novel therapeutic targets for rectal cancer.

## Materials and Methods

### Clinical Samples

Forty-six rectal cancer tissues and normal tissues were collected from patients who received treatment in the Affiliated Hospital of Youjiang Medical University for Nationalities. These tissues were snap-frozen in liquid nitrogen and stored at −80°C until usage. These patients did not receive anti-cancer treatments before enrollment. All participants provided written informed consent and the study was approved by the ethics committee of the Affiliated Hospital of Youjiang Medical University for Nationalities.

### Cells

Cells including cancer cells (SW837 and SW1463) and normal human colorectal epithelial cell line (FHC) were purchased from the Cell Bank of the Chinese Academy of Sciences (Shanghai, China). These cells were incubated in DMEM with the supplement of 10% fetal bovine serum at 37°C in a moist incubator containing 5% CO_2_.

### Transfection

The SNHG17 sequence was inserted into the NheI/XbaI sites of pcDNA3.1 by GenePharma (Shanghai, China) and named as pSNHG17. Small interfering RNA against SNHG17 (si-SNHG17, 5'-GAUUGUCAGCUGACCUCUGUCCUGU-3') and stanniocalcin 2 (si-STC2, 5'-GAAUGCUACCUCAAGCACGA-3') or miR-361-3p mimic (5'-UCCCCCAGGUGUGAUUCUGAUUU-3'), and the corresponding negative controls (si-NC, 5'-UUCUCCGAACGUGUCAGGU-3' or mi-NC, 5'-GCGUGCUUCCGAUUGUUCUGUG-3') were also bought from GenePharma. Then, they were transfected into rectal cancer cells with Lipofectamine 2000 (Thermo Fisher Scientific, Waltham, MA, United States) according to the supplier’s recommendations. After transfection for 48 h, cells were collected for analyses.

### Real-Time Quantitative PCR

Trizol reagent was used to extract total cellular RNA from tissues and cells. First strand cDNA was synthesized with the PrimerScript kit (Takara, Dalian, Liaoning, China). RT-qPCR was performed using SYBR Green (Takara) at the Applied Biosystems 7500 (Foster City, CA, United States). The Delta Delta Ct (ddCt) method was used to measure relative gene expression levels. Primers were as follows: SNHG17: 5'-GTTCCTGGGGCTTGGATGAT-3' (sense), 5'-GATCTAAGGCTGAGACCCACG-3' (antisense); STC2: 5'-TCTTGTGAGATTCGGGGCTT-3' (sense), 5'-ACAGGTCGTGCTTGAGGTAG-3' (antisense); ki-67: 5'-GAGGTGTGCAGAAAATCCAAA-3' (sense), 5'-CTGTCCCTATGACTTCTGGTTGT-3' (antisense); Bcl-2: 5'-CGACTTTGCAGAGATGTCCA-3' (sense), 5'-ATGCCGGTTCAGGTACTCAG-3' (antisense); Bax: 5'-TGCAGAGGATGATTGCTGAC-3' (sense), 5'-GAGGACTCCAGCCACAAAGA-3' (antisense); GAPDH: 5'-GTCTCCTCTGACTTCAACAGCG-3' (sense), 5'-ACCACCCTGTTGCTGTAGCCAA-3' (antisense); miR-361-3p: 5'-TCCCCCAGGTGTGATTCTGATTT-3' (sense), 5'-GCAAATCAGAATCACACCTG-3' (antisense); U6 snRNA: 5'-CTCGCTTCGGCAGCACA-3' (sense), 5'-AACGCTTCACGAATTTGCGT-3' (antisense). The RT-qPCR procedure used was: 95°C for 5 min, followed by 30 cycles at 95°C for 10s and 60°C for 30s.

### Western Blot

Cells were washed with phosphate-buffered saline (PBS) and treated with RIPA lysis buffer to extract proteins. The concentration was quantified with bicinchoninic acid (BCA) kit. Subsequently, proteins were separated at SDS-PAGE and then transferred onto the PVDF membranes. After being blocked with BSA, membranes were incubated with primary antibodies at 4°C for 12 h, followed by horseradish peroxidase (HRP)-labeled secondary antibody (ab6721). Primary antibodies were Bcl-2 (ab182858) and Bax (ab32503). The protein signal was developed with Electrochemiluminescence and analyzed with Image J software.

### Bioinformatic Analysis

The expression levels of SNHG17 and STC2 in rectal cancer tissues and normal tissues were analyzed at GEPIA.^1^ The expression level of miR-361-3p in rectal cancer tissues and normal tissues was analyzed using UALCAN.^2^ The protein levels of STC2 in rectal cancer tissues and normal tissues were analyzed using Human Protein Atlas.^3^

### Cell Counting Kit-8 Assay

Cells were plated in 96-well plates at the density of 1×10^4^ cells per well. After 24, 48, and 72 h of seeding, Cell counting kit-8 (CCK-8) reagent (Beyotime, Haimen, Jiangsu, China) was added to the plate for another 2 h. The optical density of each well was measured at the wavelength of 450 nm.

### Flow Cytometry Assay

Cells were collected and treated with trypsin to detect cell apoptosis rate using flow cytometric analysis. Then, cells were suspended in a binding buffer and double-stained with Annexin V-FITC and PI (Beyotime) for 15 min in the dark according to the supplier’s instructions. After that, cells were collected to detect cell apoptosis rates using flow cytometry (BD Biosciences, San Jose, CA, United States).

### Luciferase Reporter Assay

The ENCORI website was used to predict the miRNA targets of SNHG17 and also the messenger RNA target of miRNA. After prediction, miR-519c-3p and STC2 were selected for the following analyses with the following criteria: (1) the miRNA expression level was downregulated, while miRNA target gene expression level was upregulated in tumor tissues; and (2) that targets have roles in carcinogenesis based on previous studies. The 3'-UTR of wild-type sequences of SNHG17 or STC2 (wt/mt-SNHG17/STC2) were inserted into pmiR-GLO (Promega, Beijing, China). Then, the vectors were transfected into rectal cancer cells along with miRNAs using Lipofectamine 2000. After transfection for 48 h, relative luciferase activity was measured with the Dual-luciferase activity reporter system (Promega).

### RNA Pull-Down Assay

RNA pull-down assay was conducted to confirm the interaction of SNHG17 and miR-361-3p. The wild-type or mutant sequence of miR-361-3p was labeled with biotin using Biotin RNA labeling Mix (Ambio Life). Then, biotin-wt-miR-361-3p, biotin-mt-miR-361-3p, or biotin-mi-NC were incubated with rectal cancer cell lysates. Finally, beads were washed with buffer and then treated with Trizol to extract the RNA sample. The relative gene expression level was measured with the RT-qPCR method.

### RNA Immunoprecipitation Assay

RNA immunoprecipitation (RIP) assay was conducted to confirm the interaction of miR-361-3p and STC2 using Magna RIP Kit (Millipore, Billerica, MA, United States) according to the provided instructions. Cells were treated with lysis buffer and then incubated with beads conjugated with anti-Argonaute2 (anti-Ago2) or anti-IgG antibody. After being treated with proteinase K, the RNA sample in the bead was isolated with Trizol and then analyzed using the RT-qPCR method to detect relative gene expression levels.

### Animal Experiment

BALB/c nude mice were divided into two groups. Rectal cancer cells with sh-SNHG17 or sh-NC stable transfection were injected into the flanks of the mouse. The tumor size was calculated every 7 days by measuring the width and length of the tumor. The formula used to calculate tumor size was length × width^2^/2. After 4 weeks, mice were sacrificed to obtain the tumor tissues and then weighed. The animal study protocol was approved by the ethics committee of the Affiliated Hospital of Youjiang Medical University for Nationalities.

### Exploration SNHG17, miR-361-3p, and STC2 Expression Level in RC Using Online Available Datasets

To explore the levels of SNHG17, miR-361-3p, and STC2 in RC tissues and normal tissues, we searched and downloaded several datasets containing sequencing data from cancer and noncancerous tissues from RC patients from Gene Expression Omnibus.^4^

### Statistical Analysis

GraphPad software was used to analyze the data obtained from these experiments and then presented as mean ± SD. The significance in groups was analyzed with Student’s *t*-test or ANOVA. *p* < 0.05 was considered statistically significant.

## Results

### SNHG17 Is Upregulated Expression in Rectal Cancer

**Figure 1:**
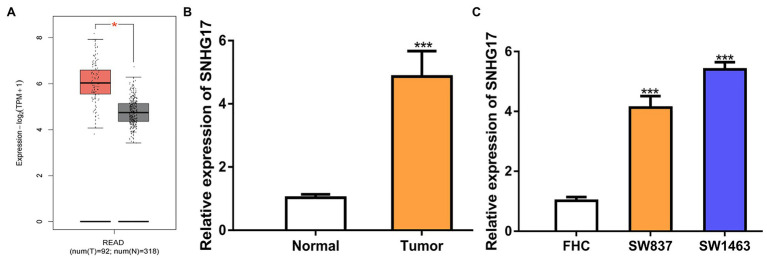
SNHG17 expression is increased in rectal cancer. **(A)** Level of SNHG17 in rectal cancer tissues and normal tissues was analyzed at GEPIA. **(B)** Level of SNHG17 in rectal cancer tissues and normal tissues was analyzed by RT-qPCR. **(C)** Level of SNHG17 in rectal cancer cells and normal cells was analyzed by RT-qPCR. ^***^p < 0.001; ^*^p < 0.05. SNHG17, small nucleolar RNA host gene 17; RT-qPCR, real-time quantitative PCR.

To evaluate the involvement of SNHG17 in rectal cancer progression, the expression level of rectal cancer tissues and normal tissues was analyzed using TCGA data, GEO data, and our cohorts. As shown in Figure 1A, SNHG17 was significantly elevated in rectal cancer tissues compared with normal tissues *via* analyzing TCGA data (*p* < 0.05). The analysis of GEO data also indicated that SNHG17 increased expression in rectal cancer (Supplementary Figure 1A, *p* < 0.01). Additionally, we observed increased SNHG17 expression levels in the rectal cancer tissues (4.86 ± 0.81) we collected compared with normal tissues (1.03 ± 0.11; Figure 1B, *p* < 0.001). Moreover, RT-qPCR analysis showed that SNHG17 increased expression in rectal cancer cells including SW837 (4.13 ± 0.38) and SW1463 (5.40 ± 0.25) compared with normal human colorectal epithelial cell line FHC (1.01 ± 0.13; Figure 1C, *p* < 0.001).

### Downregulation of SNHG17 Inhibits Rectal Cancer Cell Proliferation and Induces Cell Apoptosis

**Figure 2:**
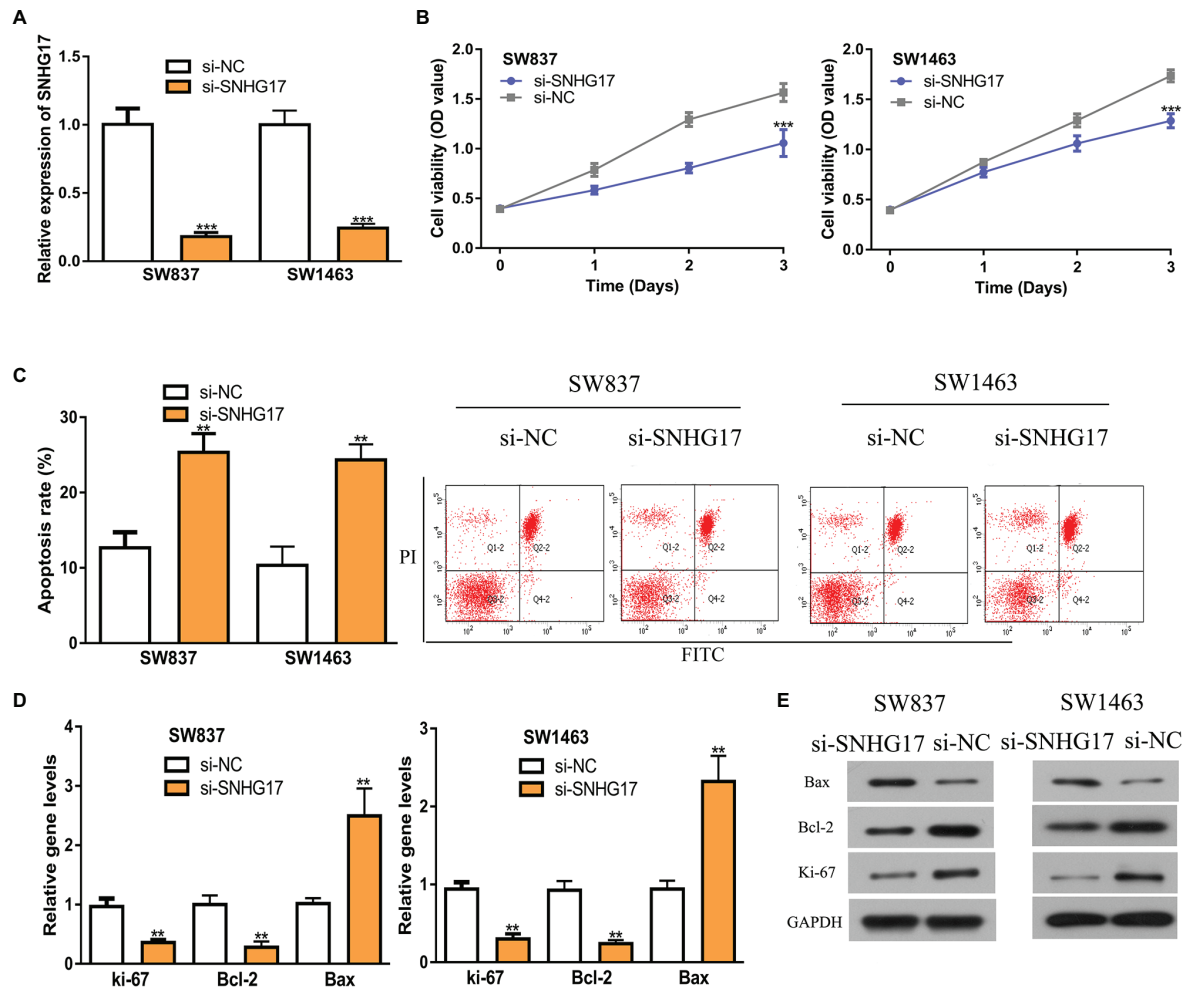
Down-regulation of SNHG17 inhibits the malignant behavior of rectal cancer cells. **(A)** Level of SNHG17 in rectal cancer cells transfected with si-NC or si-SNHG17 was analyzed by RT-qPCR. **(B)** Depletion of SNHG17 decreased the proliferation of rectal cancer cells. **(C)** Flow cytometry analysis was performed to evaluate the apoptosis of rectal cancer cells following si-NC or si-SNHG17 transfection. **(D)** Ki-67, Bcl-2, and Bax gene expression levels in rectal cancer cells following si-NC or si-SNHG17 transfection. **(E)** Ki-67, Bcl-2, and Bax protein expression levels in rectal cancer cells following si-NC or si-SNHG17 transfection. ^***^p < 0.001; ^**^p < 0.01. SNHG17, small nucleolar RNA host gene 17; RT-qPCR, real-time quantitative PCR; si-SNHG17, small interfering RNA against SNHG17; si-NC, negative control siRNA.

Since SNHG17 was increased in rectal cancer cells, we downregulated its expression with si-SNHG17. RT-qPCR confirmed that the transfection efficiency of si-SNHG17 (Figure 2A, 1.00 ± 0.12 vs. 0.18 ± 0.03 in SW837, 1.00 ± 0.10 vs. 0.24 ± 0.03 in SW1463, *p* < 0.001). The effects of SNHG17 knockdown on rectal cancer cell proliferation were measured with CCK-8 assay. The results shown in Figure 2B indicate that the depletion of SNHG17 suppresses rectal cancer cell proliferation (1.06 ± 0.14 vs. 1.57 ± 0.09 in SW837, 1.29 ± 0.07 vs. 1.74 ± 0.06 in SW1463, *p* < 0.001). Meanwhile, flow cytometry assay demonstrated that SNHG17 knockdown stimulates rectal cancer cell apoptosis (Figure 2C, 12.67 ± 2.08 vs. 25.33 ± 2.52 in SW837, 10.33 ± 2.52 vs. 24.33 ± 2.08 in SW1463, *p* < 0.01). We then analyzed several biomarkers for proliferation and apoptosis *via* the RT-qPCR and western blot methods. We showed that ki-67 and Bcl-2 expression level were decreased, while Bax was increased in rectal cancer cells after SNHG17 knockdown (Figures 2D,E, *p* < 0.01).

### SNHG17 Sponges miR-361-3p in Rectal Cancer

**Figure 3:**
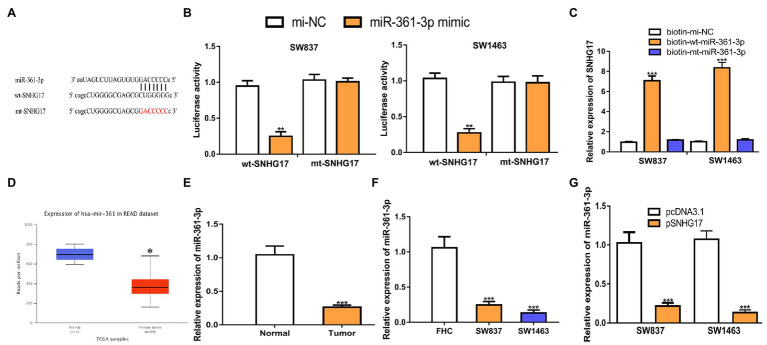
SNHG17 sponges miR-361-3p in rectal cancer cells. **(A)** The predicted binding site of SNHG17 at miR-361-3p. **(B)** Luciferase reporter assay was performed in rectal cancer cells showed transfection of miR-361-3p mimic decreased the relative luciferase activity of cells harboring wt-SNHG17. **(C)** RNA pull-down assay illustrated that SNHG17 could interact with miR-361-3p. **(D)** Level of miR-361-3p in rectal cancer tissues and normal tissues was analyzed at UALCAN. **(E)** Level of miR-361-3p in rectal cancer tissues and normal tissues was analyzed by RT-qPCR. **(F)** Level of miR-361-3p in rectal cancer cells and normal cells was analyzed by RT-qPCR. **(G)** Level of miR-361-3p in rectal cancer cells with pcDNA3.1 or pSNHG17 was analyzed by RT-qPCR. ^***^p < 0.001; ^**^p < 0.01; *p < 0.05. SNHG17, small nucleolar RNA host gene 17; RT-qPCR, real-time quantitative PCR; miR-361-3p, microRNA-361-3p; wt, wild-type; mt, mutant.

Increasing evidence showed that lncRNA serves as a sponge for miRNA, and thus affects target gene expression. To explore the mechanisms of SNHG17 in rectal cancer, we analyzed its miRNA target and a binding region between SNHG17 and miR-361-3p was observed (Figure 3A). To confirm this prediction, luciferase activity was conducted and found that miR-361-3p overexpression significantly suppressed the luciferase activity of wt-SNHG17 (0.95 ± 0.08 vs. 0.25 ± 0.06 in SW837, 1.03 ± 0.08 vs. 0.27 ± 0.06 in SW1463, *p* < 0.01) but not that of mt-SNHG17 (1.03 ± 0.08 vs. 1.00 ± 0.05 in SW837, 0.98 ± 0.08 vs. 0.97 ± 0.10 in SW1463; Figure 3B). RNA pull-down assay indicated that SNHG17 was enriched by bio-wt-miR-361-3p (Figure 3C, 0.95 ± 0.08 vs. 7.07 ± 0.47 in SW837, 1.00 ± 0.05 vs. 8.35 ± 0.55 in SW1463, *p* < 0.001). Furthermore, we showed miR-361-3p levels were decreased in rectal cancer tissues and cells compared with normal counterpart in TCGA data (Figure 3D, *p* < 0.05), GEO data (Supplementary Figure 1B, *p* < 0.01), our cohorts (Figure 3E, 1.04 ± 0.13 vs. 0.26 ± 0.03, *p* < 0.001), and cells (Figure 3F, 1.06 ± 0.16 in FHC vs. 0.25 ± 0.05 in SW837 vs. 0.13 ± 0.04 in SW1463, *p* < 0.001). To evaluate the interaction of SNHG17 and miR-361-3p, we showed that the overexpression of SNHG17 decreased the miR-361-3p expression level in rectal cancer cells (Figure 3G, 1.03 ± 0.14 vs. 0.22 ± 0.04 in SW837, 1.07 ± 0.11 vs. 0.13 ± 0.04 in SW1463, *p* < 0.001).

### STC2 Is a Target of miR-361-3p

**Figure 4:**
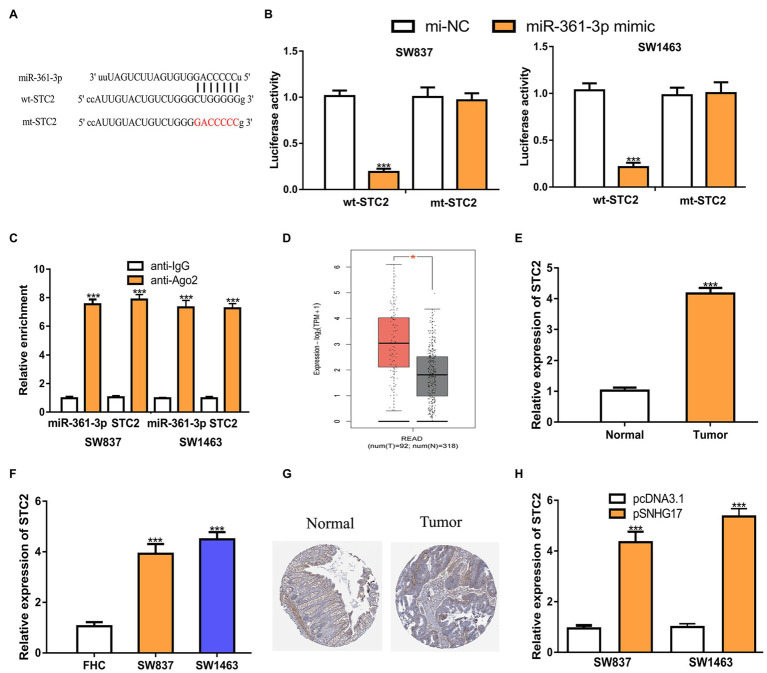
STC2 is a target of miR-361-3p in rectal cancer cells. **(A)** Alignment of the miR-361-3p seed sequence within the 3'-UTR of STC2. **(B)** Luciferase reporter assay was performed in rectal cancer cells showed transfection of miR-361-3p mimic decreased the relative luciferase activity of cells harboring wt-STC2. **(C)** RIP assay illustrated that STC2 could interact with miR-361-3p. **(D)** Level of STC2 in rectal cancer tissues and normal tissues was analyzed at GEPIA. **(E)** Level of STC2 in rectal cancer tissues and normal tissues was analyzed by RT-qPCR. **(F)** Level of STC2 in rectal cancer cells and normal cells was analyzed by RT-qPCR. **(G)** STC2 protein expression level in rectal cancer tissues and normal tissues was analyzed at Human Protein Atlas. **(H)** Level of STC2 in rectal cancer cells with pcDNA3.1 or pSNHG17 was analyzed by RT-qPCR. ^***^p < 0.001. SNHG17, small nucleolar RNA host gene 17; RT-qPCR, real-time quantitative PCR; miR-361-3p, microRNA-361-3p; wt, wild-type; mt, mutant; STC2, stanniocalcin 2; RIP, RNA immunoprecipitation.

As the functions of miRNAs rely on modulating target gene expression, we predicted the targets of miR-361-3p. STC2 was predicted as a target of miR-361-3p (Figure 4A). Luciferase activity assay showed that the overexpression of miR-361-3p reduces the luciferase activity of cells expressing wt-STC2 (Figure 4B, 1.01 ± 0.06 vs. 0.19 ± 0.04 in SW837, 1.03 ± 0.07 vs. 0.21 ± 0.05 in SW1463, *p* < 0.001). RIP assay confirmed the co-enrichment of miR-361-3p and STC2 (Figure 4C, *p* < 0.001). The analysis of STC2 expression in rectal cancer tissues and cells showed that STC2 increased expression in tumor tissues and cells (Figures 4D–F; Supplementary Figure 1C). The analysis of STC2 expression in rectal cancer tissues and normal tissues using the IHC method and showed that the STC2 protein level was also increased in rectal cancer tissues compared with normal tissues (Figure 4G). In addition, we found that increased SNHG17 expression could stimulate STC2 expression levels in rectal cancer cells (Figure 4H, 0.95 ± 0.12 vs. 4.35 ± 0.41 in SW837, 1.00 ± 0.13 vs. 5.36 ± 0.31 in SW1463, *p* < 0.001).

### STC2 Knockdown Abolishes the Effects Caused by SNHG17 Overexpression

**Figure 5:**
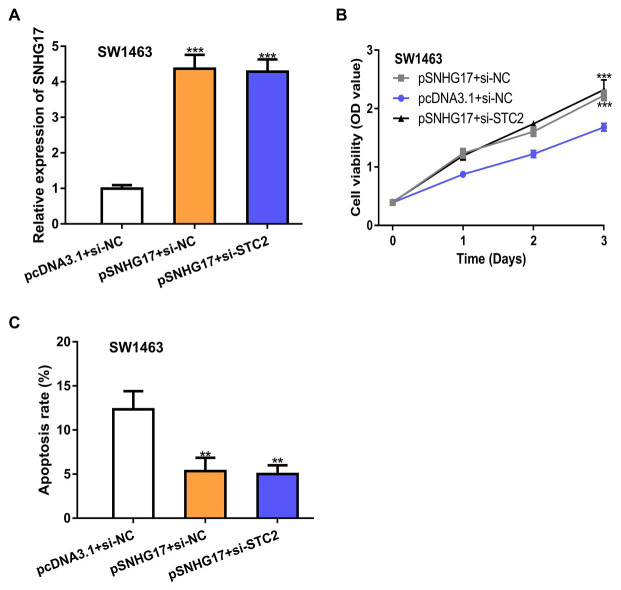
STC2 knockdown abolishes the effects caused by pSNHG17 in SW1463 cells. **(A)** Level of SNHG17 in rectal cancer cells transfected with pcDNA3.1+si-NC, pSNHG17+si-NC, or pSNHG17+si-STC2 was analyzed by RT-qPCR. **(B)** Cell proliferation assay to detect cell proliferation rate with pcDNA3.1+si-NC, pSNHG17+si-NC, or pSNHG17+si-STC2. **(C)** Flow cytometry analysis was performed to evaluate the apoptosis of rectal cancer cells following pcDNA3.1+si-NC, pSNHG17+si-NC, or pSNHG17+si-STC2 transfection. ^***^p < 0.001; ^**^p < 0.01. SNHG17, small nucleolar RNA host gene 17; RT-qPCR, real-time quantitative PCR; si-STC2, small interfering RNA against STC2; si-NC, negative control siRNA; STC2, stanniocalcin 2.

To explore whether STC2 was a functional target for SNHG17, rectal cancer cells were co-transfected with pSNHG17 and si-STC2. Firstly, we showed that pSNHG17 increased SNHG17 expression, while si-STC2 has little influence on SNHG17 expression (Figure 5A, *p* < 0.001). In the CCK-8 assay, the knockdown of STC2 reversed the stimulation effects of SNHG17 on rectal cancer cell proliferation (Figure 5B, *p* < 0.001). The silence of STC2 also suppressed cell apoptosis and abolished the effect of pSNHG17 on cell apoptosis (Figure 5C, *p* < 0.01).

### Knockdown of SNHG17 Suppresses Rectal Cancer Tumor Growth

**Figure 6:**
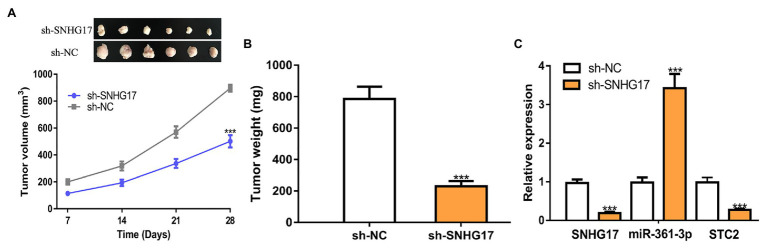
SNHG17 knockdown inhibits tumor xenograft growth. **(A)** Tumor volume, **(B)** Tumor size, and **(C)** Expression of SNHG17, miR-361-3p, and STC2 in nude mice with sh-SNHG17 or sh-NC transfection. ^***^p < 0.001. SNHG17, small nucleolar RNA host gene 17; miR-361-3p, microRNA-361-3p; STC2, stanniocalcin 2; sh-SNHG17, short hairpin RNA against SNHG17; sh-NC, negative control shRNA.

We further analyzed the biological roles of SNHG17 *in vivo*. In our work, rectal cancer cells transduced with sh-SNHG17 were injected into nude mice. As shown in Figures 6A,B, tumor volume (897 ± 66 vs. 502 ± 46, *p* < 0.001) and tumor weight (785 ± 79 vs. 230 ± 34, *p* < 0.001) were significantly reduced by sh-SNHG17. Analysis of gene expression showed that SNHG17 (0.97 ± 0.09 vs. 0.19 ± 0.04, *p* < 0.001) and STC2 was decreased (0.98 ± 0.13 vs. 0.27 ± 0.04, *p* < 0.001), which miR-361-3p (0.98 ± 0.14 vs. 3.42 ± 0.37, *p* < 0.001) was increased by sh-SNHG17 (Figure 6C).

## Discussion

The initiation and progression of rectal cancer are multiple carcinogenesis processes that involve numerous genetic alterations and biological process changes (Hanahan and Weinberg, 2011). It is now clear that RNA is not only just a messenger between DNA and proteins but also can regulate various cellular processes. ncRNAs represent about 98% of all human transcripts, which are much higher than the numbers of protein-coding transcripts (Boeke et al., 2016). Recent studies have begun to explore the functions and mechanisms of ncRNAs in cancers (Kung et al., 2013; Anastasiadou et al., 2018).

In this study, lncRNA SNHG17 was found to elevate expression in both rectal cancer tissues and cells compared with normal tissues and cells. The results revealed that SNHG17 could function as an oncogenic lncRNA and that it is highly expressed in cancers including tongue squamous cell carcinoma and breast cancer (Du et al., 2020; Liu et al., 2020). SNHG17 overexpression was revealed to stimulate cancer progression by affecting cell growth and other cell behaviors (Du et al., 2020; Liu et al., 2020). Considering that SNHG17 are overexpressed in rectal cancer, this work silenced SNHG17 expression. We found that the knockdown of SNHG17 suppresses cell proliferation by stimulating cell apoptosis, indicating the involvement of SNHG17 in regulating the malignant behaviors of rectal cancer cells. Analyses of the proliferation and apoptosis markers using RT-qPCR and western blot methods confirmed the results of the CCK-8 assay and flow cytometry assay. Importantly, we revealed that the knockdown of SNHG17 could suppress rectal cancer tumor growth *in vivo*. These results indicate the oncogenic role of SNHG17 in regulating rectal cancer progression.

In 2011, a ceRNA theory by Salmena et al. (2011) revealed the mechanisms of ncRNAs in regulating cellular functions. The key to this theory is that ncRNA can compete with protein-coding genes to bind with miRNA to affect target protein-coding gene expression (Salmena et al., 2011). Here, we explore the potential mechanisms of SNHG17 in rectal cancer. The present study showed that miR-361-3p was a target of SNHG17 by luciferase activity reporter assay and RNA pull-down assay. miR-361-3p was revealed to have controversial roles in affecting cancer progression (Hua et al., 2020; Qi and Li, 2020). For example, a recent study showed that miR-361-3p can stimulate breast cancer progression (Hua et al., 2020). On the contrary, miR-361-3p was found to be decreased by lncRNA PVT1 in non-small cell lung cancer to affect cancer cell growth and metastasis (Qi and Li, 2020). Furthermore, we analyzed the targets of miR-361-3p and found that STC2 was a target of miR-361-3p with luciferase activity reporter assay and RIP assay. Ago2 is reported to be a crucial component of the RNA-induced silencing complex (RISC) and can be used to detect genes targets by miR-361-3p (Yu et al., 2018). It has been reported that STC2 promotes nasopharyngeal carcinoma cell migration and invasion and that it is an oncogene (Lin et al., 2014). Another work showed that STC2 was associated with head and neck squamous cell carcinoma cell proliferation through GSEA analysis (Ma et al., 2020). Here, we also revealed that the high expression status of STC2 in rectal cancer, and the knockdown of STC2 can suppress rectal cancer cell proliferation. Next, we showed that STC2 was a functional target of SNHG17 as STC2 depletion can reverse the effects of SNHG17 overexpression on rectal cancer cell proliferation. The present study can be further advanced by performing RNA-seq in cells with STC2 expression level alterations. Furthermore, the biological pathways altered after STC2 expression alteration can be identified to further explore the signaling pathways involved in this process.

Collectively, we explored the roles and mechanisms of SNHG17 in rectal cancer and discovered that SNHG17 upregulated expression in rectal cancer tissues and cells. Functionally, SNHG17 can affect rectal cancer tumor growth *in vitro* and *in vivo* by regulating the miR-361-3p/STC2 axis. The new regulatory axis identified in this work can help us to understand the pathogenesis process of rectal cancer.

## Data Availability Statement

The original contributions presented in the study are included in the article/Supplementary Material, further inquiries can be directed to the corresponding author.

## Ethics Statement

The studies involving human participants were reviewed and approved by Ethics Committee of Affiliated Hospital of Youjiang Medical University for Nationalities. The patients/participants provided their written informed consent to participate in this study. The animal study was reviewed and approved by Ethics Committee of Affiliated Hospital of Youjiang Medical University for Nationalities.

## Author Contributions

All authors listed have made a substantial, direct and intellectual contribution to the work, and approved it for publication.

### Conflict of Interest

The authors declare that the research was conducted in the absence of any commercial or financial relationships that could be construed as a potential conflict of interest.

## Footnotes

^1^http://gepia2.cancer-pku.cn/#index

^2^http://ualcan.path.uab.edu/analysis-mir.html

^3^https://www.proteinatlas.org

^4^https://www.ncbi.nlm.nih.gov/geo/

## Supplementary Material

Supplementary Figure 1 Exploration of SNHG17, miR-361-3p, and STC2 expression level in rectal cancer tissues and normal tissues using datasets obtained from the Gene Expression Omnibus. **(A)** SNHG17, **(B)** miR-361-3p, and **(C)** STC2 levels in rectal cancer tissues and normal tissues. SNHG17: small nucleolar RNA host gene 17; miR-361-3p: microRNA-361-3p; STC2: stanniocalcin 2.
