# Supplementary figures and images for: SNHG17 Serves as an Oncogenic lncRNA by Regulating the miR-361-3p/STC2 Axis in Rectal Cancer
Source: Front Genet. 2021 Jun 23;12:654686. doi: 10.3389/fgene.2021.654686 (PMC8260683; doi:10.3389/fgene.2021.654686)

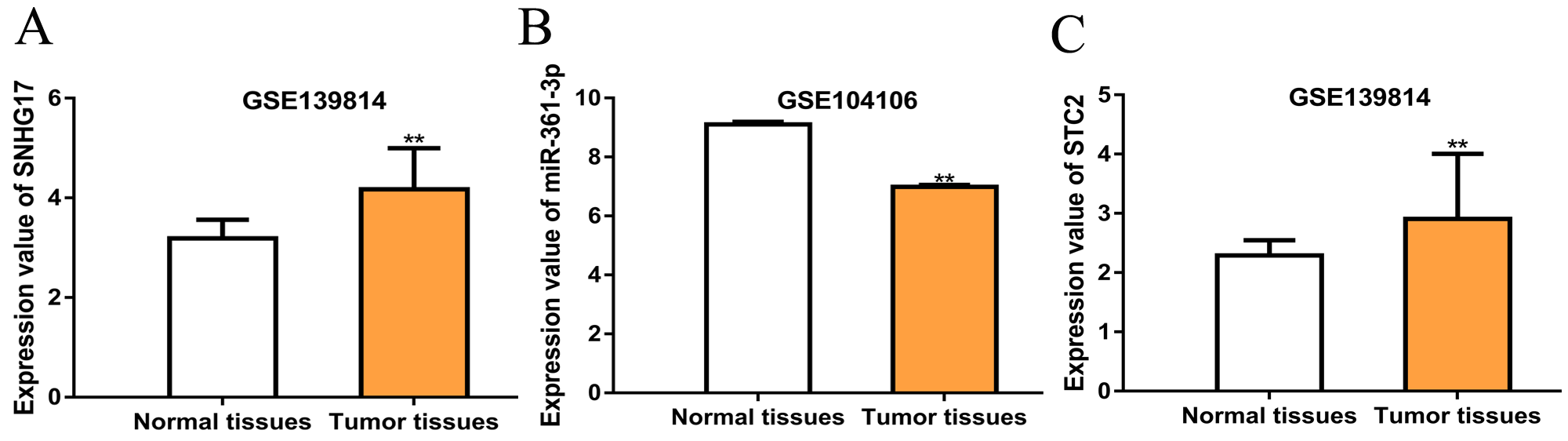

Supplement: Supplementary Figure 1 — Exploration of SNHG17, miR-361-3p, and STC2 expression level in rectal cancer tissues and normal tissues using datasets obtained from the Gene Expression Omnibus. (A) SNHG17, (B) miR-361-3p, and (C) STC2 levels in rectal cancer tissues and normal tissues. SNHG17: small nucleolar RNA host gene 17; miR-361-3p: microRNA-361-3p; STC2: stanniocalcin 2. [file Image_1.TIF]
